# Influences of Social Distancing and attachment styles on the strength of the Halo Effect

**DOI:** 10.1371/journal.pone.0256364

**Published:** 2021-08-26

**Authors:** Giulio Gabrieli, Yun Yee Lim, Gianluca Esposito

**Affiliations:** 1 Psychology Program, Nanyang Technological University, Singapore, Singapore; 2 Lee Kong Chian School of Medicine, Nanyang Technological University, Singapore, Singapore; 3 Department of Psychology and Cognitive Science, University of Trento, Trento, Italy; University Hospitals Tubingen: Universitatsklinikum Tubingen, GERMANY

## Abstract

The Halo Effect is a widely studied phenomenon that interests multiple disciplines. The relationship between Aesthetics Appearance and perceived Trustworthiness has especially gathered the attention of social scientists. While experimental works compared the strength of the Halo Effect in different situations (e.g. different genders’ faces), little is known about the stability of the Halo. During the COVID-19 pandemic, we have been urged to distance ourselves from others. Similar suggestions may alter the relationship between Aesthetic Appearance and Perceived Trustworthiness. Moreover, previous works reported that individuals’ attachment styles affected their emotional responses to the pandemic. Individuals’ attachment styles may influence the magnitude of change of the Halo. Here we investigate how priming (Social Distancing or Contact with others) affects the strength of the Halo Effect, with respect to individuals’ attachment styles. Participants (N = 298) rated the Aesthetics and Perceived Trustworthiness of strangers’ faces (N = 96) presented twice, before and after the presentation of a prime. Results revealed that individuals’ attachment styles affect the strength of the Halo. However, we found no evidence supporting the fact that different attachment styles lead to differences in the magnitude of changes after priming. Results help shed light on how attachment styles influence individuals’ impression formation.

## Introduction

The *Halo Effect* is a cognitive bias in impression formation whereby the evaluation of another single attribute affects the global evaluation of an individual’s attributes [1]. When referring to aesthetic appearance, the Halo Effect is observed when an individual’s physical appearance is used as a basis for the evaluation of other attributes that are not related to aesthetics. For example, good-looking individuals may be perceived as smart and intelligent, even though these two traits are unrelated to physical attractiveness. The Halo Effect is known to be intuitive, constant, and pervasive [2–6]. It is a widely investigated psychological phenomenon, which has received the attention of researchers in Computer Science, Empirical Aesthetics, and Social Psychology [7–11].

First proposed by Thorndike [12], the term Halo Effect is used to describe the radiating effects of a single attribute on the evaluations of other traits. Empirically observed in multiple fields, the Halo Effect has been observed in numerous domains of impression formation. Over the years, several works demonstrated the relationship between physical appearance on social warmth [13], happiness, personality [14], competence, success in marriage and other moral activities [15, 16], integrity [15], intellect [17], and many more [18].

Controversial findings have been found for what concerns the importance of rated faces’ gender. In a study conducted by Carter [19], participants—men and women—reported their first impression of a counselor, man or woman, that was either attractive or unattractive. Results of the work highlighted the presence of significant interaction effects between perceived attractiveness and gender-related variables. However, other works revealed no significant differences in the ratings given by men and women [20, 21]. While different explanations have been given, such as that in the case of Carter [19], the stereotypical image of a counselor could have played a role in shaping the results [22], few studies have attempted to address the problem with sufficient statistical power [21]. Moreover, only a limited number of studies investigated the stability of the effect over time. A previous work on the generalizability of the Halo Effect [21] serendipitously revealed that the strength of the relationship between perceived Aesthetics and perceived Trustworthiness of strangers’ faces could be influenced by external events, such as the spreading of news about the COVID-19 pandemic outbreak. Similarly, researchers focused on the relationship between specific personality traits [23] or attachment styles on individuals’ emotional reactions to the pandemic and preventive measures. The latter is of special interest for faces’ perception, given that the prominent role of attachment history on individuals’ social interactions [24]. Based on the attachment theory [25], adult individuals use their early experiences of attachment during infancy as a reference for their social relationships. Different attachment styles have been proven to affect, for example, the perception of others’ pain [26], the quality of interaction with their children [27], as well as the perception of complex emotional scenes [28].

The psychological impact of the COVID-19 outbreak has been studied in relation to individuals’ attachment styles in a work conducted by Moccia et al. [29], which demonstrated that avoidant and secure attached individuals had a lower risk of psychological burden, as compared to anxiously attached individuals. While the results for the avoidant individuals may be explained in terms of a lower difficulty in engaging in self-isolation or social distancing practices, it is also possible that avoidantly attached individuals are less likely to express their difficulties, therefore appearing calm despite feeling differently in their private sphere. Despite the strong evidence, it is unlikely that the attachment style is the only factor contributing to individuals’ perceived distress. Previous works demonstrated that attachment style plays a role in news perception [30] and social media use [31], suggesting that insecurely attached individuals are more likely to use social media sites to replace physical interactions. At the same time, they may receive higher exposure to news related to the pandemic [32] and thus experience more distress.

While recent works on the Halo Effect and the protective effects of the different attachment styles during the pandemic outbreak proved that policies to contrast the pandemic had a significant impact on individuals’ perception, previous works could not control for the level of exposure to news about the pandemic in a quantitative way, nor could they discern the impact of the pandemic outbreak from other personal events that could have affected participants’ responses.

Driven by the desire to overcome these limitations, we aim to investigate the stability of the Halo Effect under a controlled priming situation and assess the possibility that different attachment styles can increase the stability of the Halo Effect when perturbed by external events.

### Aim & hypothesis

This paper has four broad aims. The first is to confirm the presence or absence of significant differences in the strength of the Halo toward faces of different genders. While earlier works suggested that the Halo Effect is stronger for faces of the opposite gender, more recent works with more appropriate power were not able to demonstrate such differences. Additionally, previous works revealed that the age of the presented face but not the ethnicity influenced the strength of the Halo. Therefore, this work aims to verify such results on a different sample.

The second aim of this study is to investigate the relationship between the Strength of the Halo and individuals’ attachment styles. We expect anxious and preoccupied attached individuals to rely more on aesthetic appearances to make inferences about the trustworthiness of strangers’ faces, as compared to fearful or avoidant attached individuals. In fact, while the attachment style should play no role in the judgment of faces’ aesthetics appearances, lesser trust toward strangers by fearful or avoidant individuals should result in a lower correlation between aesthetics appearance and perceived trustworthiness, which is the strength of the Halo Effect.

The third aim is to investigate the stability of the Halo Effect under controlled manipulations. Hendrick and Costantini [33] demonstrated that the strength of the Halo could be reduced when participants were asked to focus on their reasoning. This suggests that the Halo Effect is more likely to emerge when individuals employ rapid, automatic, and constructive processing [33–35]. Also, mood has been proven to influence individuals’ judgment [36]. Specific moods can prime mood-congruent processes that are more likely to be employed when engaging in social judgment tasks [37, 38]. While previous works investigate changes in the strength of the Halo during the initial stages of the pandemic outbreak, ruling out individual differences on previously collected data was not possible. For this reason, we aim to compare the strength of the Halo Effect on participants’ ratings to the same face before and after the priming of either Social Distancing or Close Contact with others. These primes should elicit mood-congruent responses: the priming of Social Distancing should induce participants to further reflect on why they should trust someone, and therefore reduce rapid processing, with a consequent reduction of the Halo Effect.

The fourth and final aim of this study is to investigate whether different attachment styles influence the magnitude of the changes in the strength of the Halo Effect after a prime has been shown. Would primes be able to impact the strength of the Halo Effect significantly? Specific attachment styles may reduce or increase the magnitude of such changes.

In sum, the goal of this paper is to demonstrate the existence of significant gender differences in the strength of the Halo, and to study the changes in the strength of the Effect under a priming condition, considering the possible protective effects of specific attachment styles. To do so, we will address four specific hypotheses:

*H_1_*: The strength of the Halo Effect, measured as the Pearson’s correlation between Aesthetics and Trustworthiness, is stronger for opposite gender faces, as compared to same-gender faces.
*H_2_*: The strength of the Halo Effect, measured as the Pearson’s correlation between Aesthetics and Trustworthiness, is stronger for Secure and preoccupied attached individuals, as compared to fearful or avoidant attached individuals
*H_3_*: The priming of close social interactions would increase the strength of the Halo Effect, while priming of social distancing results in a decrease of the strength of the Halo Effect, exclusively toward adults’ faces, while no changes are expected after priming in the ratings of children’s faces.
*H_4_*: The magnitude of changes in the strength of the Halo Effect after priming is influenced by individuals’ attachment styles.

## Materials and methods

### Participants

To estimate the number of participants required to test our hypotheses with the proposed methodology, a power analysis has been performed using G*Power [39]. Considering *α* = 0.05, a medium effect size (f = 0.15 estimated from previous studies), one hundred forty-six (N = 146) participants are required to achieve sufficient power (power = 0.95). To take into account cross-culture differences, data from both Asians-Chinese and White participants were collected, doubling the number of estimated participants to two hundred ninety-two (N = 292) participants, balanced by ethnic group (about 146 Asians-Chinese and about 146 Whites).

Participants were recruited through the research participation system of the Nanyang Technological University (SONA), different social media—including Facebook, Twitter, and Instagram—, online communities (e.g. the “samplesize” subreddit), and online recruitment systems, such as Amazon’s MTurk. Participants recruited using SONA received research credits, while participants who participated through MTurk received monetary compensation for their time. Data collection took place between August 2020 and January 2021. Inclusion criteria were the ability to understand written English instructions, and being of legal age in the country of residence. Of the 304 participants who completer our survey, 6 participants were excluded for not identifying as either Men or Women, or for their preference not to disclose the information. Additionally, 9 participants were excluded for not identifying as either White or Asian. Therefore, the final dataset consists of the data of two hundred eighty-nine (N = 289, Mean Age = 28.96 ± 12.02, 186 Women) participants. For what concerns the ethnic background, 144 identified as Asian/Chinese (Mean Age = 20.48 ± 2.62, 112 Women), while 145 identified as White (Mean Age = 37.38 ± 11.80, 74 Women). Of the participants, 143 received university credits, while 135 received monetary compensation. A summary of participants’ demographics is reported in Table 1.

**Table 1:** Participants’ demographic information.

Ethnicity	Gender	N	Age
Asian	Men	32	22.8 ± 4.19
	Women	112	19.8 ± 1.37
White	Men	71	35.5 ± 10.06
	Women	74	39.20 ± 13.08

### Experimental procedure

The experimental paradigm, estimated number of required participants, hypotheses, and analysis were preregistered on the Open Science Framework (https://osf.io/rzdxh), and approved by the Institutional Review Board of the Nanyang Technological University.

**Fig 1:**
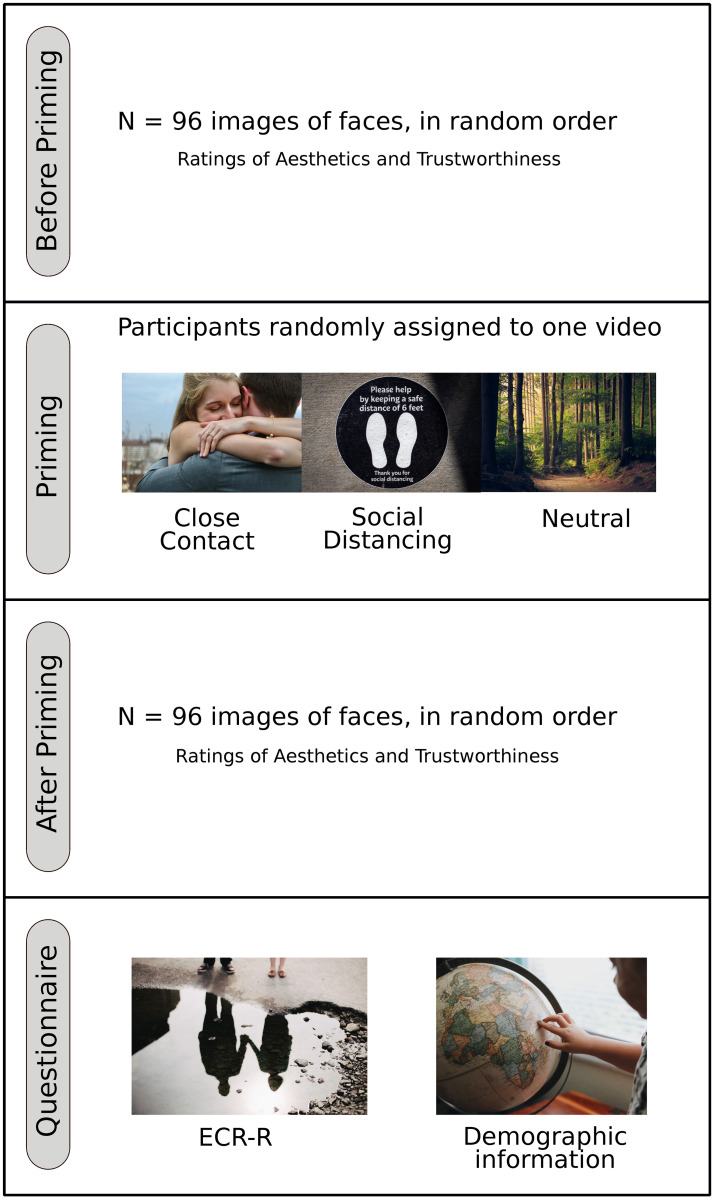
Visual representation of the experimental paradigm.

After a brief presentation of the study and after signing the informed consent, participants are presented with two blocks composed of ninety-six (N = 96 faces), presented in random order, depicting individuals of different Age (child, adult, or elderly), Gender (men or women), and Ethnicity (Asian or White). For each combination of age, gender, and ethnicity, eight (N = 8) different faces are presented. After the presentation of a face, participants were asked to rate both the Aesthetic appearance and the Perceived Trustworthiness of the presented face. No time constraints were given. For each face, the two measures will be recorded using a 100 point Likert Scale, where 0 stands for *“Not at all”*, while 100 indicates *“Extremely”*. Between the two blocks, participants were presented with a video that either promotes close contact with other individuals, a video that promotes social distancing, or a video with a natural scene (neutral condition). The three videos are matched in length. Additionally, each block contains three attention checks to verify participants’ attention to the task (e.g. *“Based on the text below, what is your favorite food? When asked for your favorite food, please select pizza.”*). The procedure, visually represented in Fig 1, has been implemented in an online questionnaire that participants could complete with no time constraints. To test the hypotheses presented in the previous section, a Multiple Linear Regression is employed with the differences in strength of the Halo Effect—measured as the Pearson’s correlation between Aesthetics and Trustworthiness judgments—before and after priming as a dependent variable. Scores on the attachment style questionnaire, priming condition, and demographic information of presented faces (Age, Gender, and Ethnicity) are measured as independent variables. All data were proved for normality of distribution by Shapiro-Wilk test with subsequent uses of non-parametric (Mann-Whitney U-test) statistics.

### Materials

#### Attachment style

Participants’ attachment styles are measured using the “Experience in Close Relationships—Revised” (ECR-R) [40], a 36-item self-report questionnaire that has proved to provide highly stable indicators of latent attachment [41]. Results of the questionnaire are indicative of individuals’ attachment status towards their partner. From the items, it is possible to obtain two indexes of participants’ attachment styles, which are called “Avoidance” and “Anxiety”. Low scores on both scales are indicative of a fairly secure attachment style, while scores high on both scales indicate that the individual presents a fearful attachment style. A low score on the anxiety scale, paired with a high score on the avoidance scale is representative of a dismissing attachment style, while the opposite situation—which is a high score on the anxiety score and a low score on the avoidance score—is indicative of a preoccupied attachment style.

#### Stimuli

Front-facing images of faces (N = 96) were selected from the FFHQ Dataset [42], a dataset containing 70,000 high-quality (1024×1024) images published on Flickr, an online photo management, and sharing tool, under different creative commons and public domain licenses (Creative Commons BY 2.0, Creative Commons BY-NC 2.0, Public Domain Mark 1.0, Public Domain CC0 1.0, or U.S. Government Works license). The dataset itself is released under the Creative Commons BY-NC-SA 4.0 license by NVIDIA Corporation and has been successfully used in previous publications on the application of Machine Learning and Neural Networks [42–45], as well as on studies on the Halo Effect [21]. Sixty-four (N = 64) stimuli of Men and Women Adults and Children of Asian and White ethnicity were drawn from a previous work on the Halo Effect [21]. In addition, thirty-two (N = 32) images of Men and Women Elderly of Asian and White ethnicity were manually selected from the FFHQ dataset and included in the stimuli set. To be included, faces had to be completely shown within the image, front-facing, and with no external elements that may affect perception (e.g. presence of other individuals in the background, animals in the frame, hats, etc.). Details about the estimation of the required number of stimuli are reported in Section Power Analysis.

For what concerns the priming videos—one promoting Social Distancing, one promoting Close Contacts with others, and a Neutral video—, we have employed three videos available on Youtube, matched in length and video quality. The video promoting Social Distancing is an instructional video by BBC news providing information about the importance and effectiveness of social distancing. The video promoting Close Contacts is drawn from a Social Experiment in which couples, adults Asian and Whites, are asked to hug each other for four minutes. Finally, the neutral video consists of sights of natural sceneries (such as lakes and mountains). Despite not being the optimal solution, using available videos was a necessity. With the current policies, we were unable to register videos promoting Close Contacts with others ourselves.

## Results

A Multiple Linear Regression (MLR) analysis was conducted to verify our four hypotheses. The effects and interaction we are interested in to verify our hypotheses are reported in Table 2, while the full results are reported in the S1 Table. The variable Gender indicates the relation between Participants’ and each Stimulus’ gender (Same vs Different gender). In an analog way, the Ethnicity variable indicates the relation between Participants’ and each Stimulus’ Ethnicity (Same vs Different Ethnicity), while the variable Age indicates the Age group of the presented face (Children, Adult, Elder). The variable Attachment Style refers to the type of attachment measured with the ECR-R. Finally, the variable Priming and Time refer to the prime received (Close Contact, Social Distancing, Neutral) and to the moment in which each data point was collected, which can be before or after the presentation of the Prime.

**Table 2:** Selected effects and interactions of the Multiple Linear Regression results’ table.

	Coeff	Std. Error	t	p-value	95% CI
Age	0.0357	0.009	4.117	**0.000** ***	[0.019; 0.053]
Gender	-0.0012	0.007	-0.169	0.866	[-0.015; 0.013]
Ethnicity	0.0016	0.007	0.227	0.820	[-0.012; 0.015]
Attachment Style	0.0096	0.004	2.224	**0.026** *	[0.001; 0.018]
Priming	0.0010	0.009	0.116	0.908	[-0.016; 0.018]
Time	0.0141	0.007	2.006	**0.045** *	[0.000; 0.028]
Age × Time	-0.0066	0.009	-0.756	0.450	[-0.024; 0.010]
Age × Priming × Time	0.0043	0.011	0.397	0.691	[-0.017; 0.025]
Priming × Time × Attachment Style	0.0026	0.005	0.497	0.619	[-0.008; 0.013]

* p < 0.05,

*** p < 0.001

**Fig 2:**
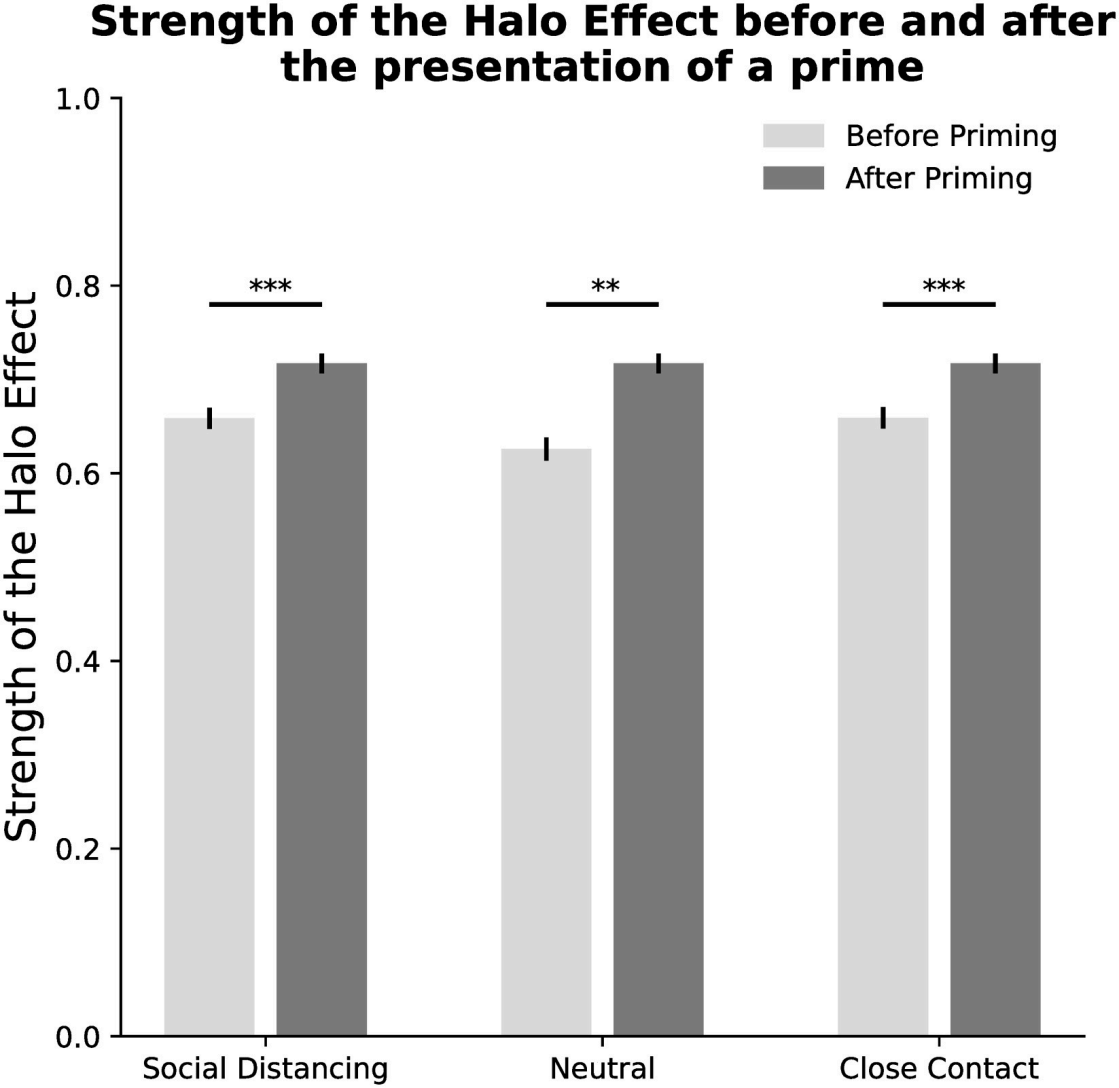
Strength of the Halo Effect, measured as Pearson’s correlation (mean and standard error) between Aesthetics and Trustworthiness ratings, by condition. ** p < 0.01, *** p < 0.001.

For our first hypothesis to be verified, which is that faces of the opposite gender present a stronger Halo Effect as compared to same-gender faces, we should see a significant main effect of Gender in our MLR. As reported in Table 2, we found no significant main effect of the relationship between stimuli and participants’ gender (indicated as Gender in Table 2) on the strength of the Halo Effect (t = -0.169, p = 0.866). Limiting to the data collected before the presentation of any prime highlighted the absence of significant differences even before any prime is presented (t = 0.0163, Corrected p = 1.00). Because of the non-normal distribution of Halo data, Mann-Whitney U tests are employed for post-hoc comparisons. Despite the absence of significant differences before the presentation of a prime, significant differences were found in the ratings given to both same (Mann–Whitney U = 1266419.0, Corrected p = 3.208 ⋅ 10^−5^) and different gender faces (Mann–Whitney U = -3.556, Corrected p = 0.00153) before and after the presentation of a prime, with a significantly stronger Halo Effect for both same and different gender faces after priming, as compared to before priming. A further focus on the difference after priming (Fig 2), a post-hoc comparison by mean of a Bonferroni corrected Mann–Whitney U test revealed that the difference in the strength of the Halo is significant when participants were exposed to a prime that promotes Social Distancing (Mann–Whitney U = 568403.0, Corrected p = 1.126 ⋅ 10^−6^), a neutral prime (Mann–Whitney U = 502560.5, Corrected p = 1.503 ⋅ 10^−3^) or to a video that promotes close contact with others (Mann–Whitney U = 595605.5, Corrected p = 1.540 ⋅ 10^−4^).

**Fig 3:**
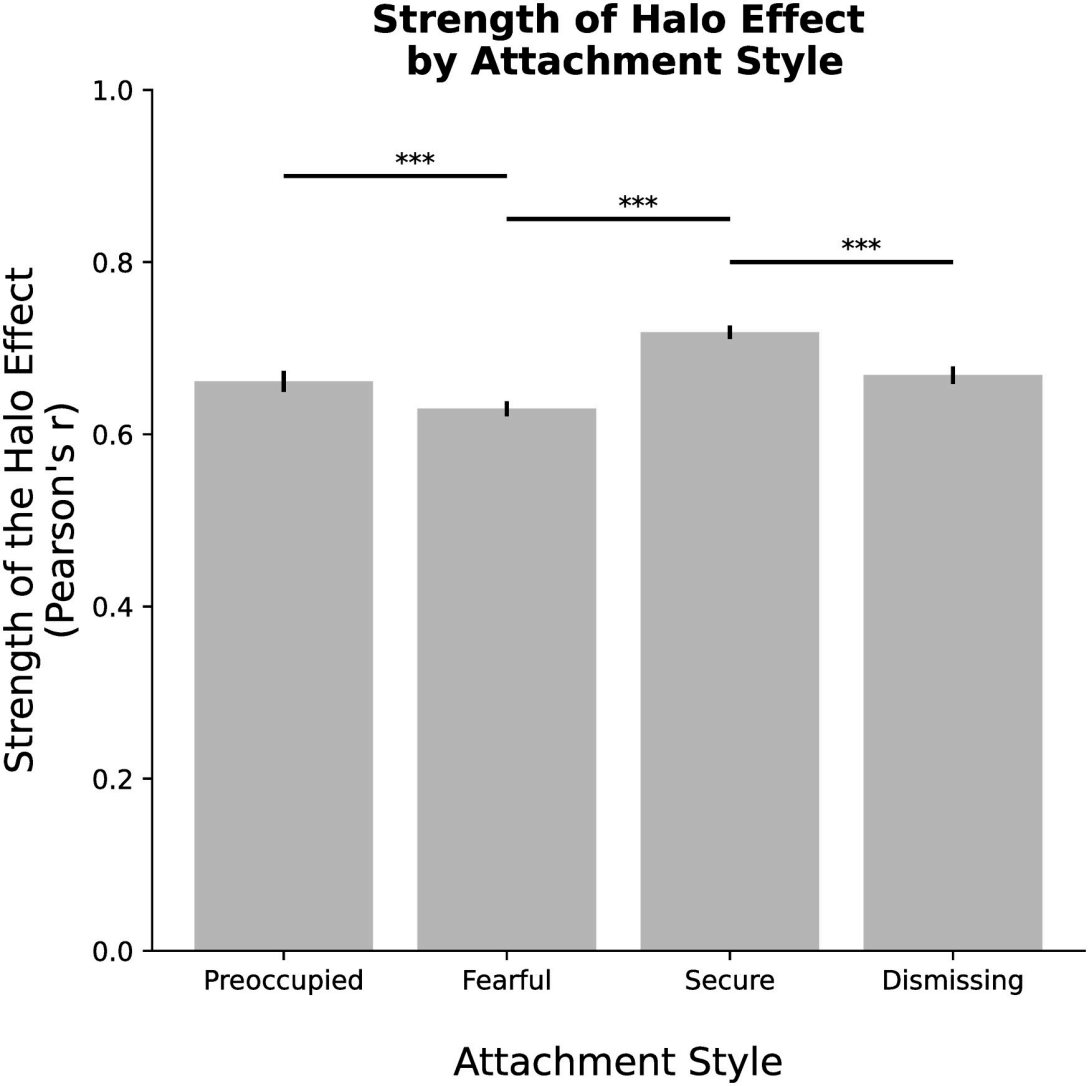
Strength of the Halo Effect, measured as Pearson’s correlation between Aesthetics and Trustworthiness ratings, by participants’ attachment style. ** p < 0.01, *** p < 0.001.

To assert that our second hypothesis H_2_ is verified, we should see a significant effect of attachment style on Halo. Results of MLR (Table 2), highlight the existence of a significant main effect of Attachment style (t = 2.224, p = 0.026) on the strength of the Halo Effect. Post-hoc analysis revealed that the strength of the Halo Effect for Secure individuals is significantly stronger than the Halo Effect of both Fearful (Mann–Whitney U = 1879919.5, Corrected p = 1.567 ⋅ 10^−11^) and Dismissing (Mann–Whitney U = 1144806.5, Corrected p = 1.067 ⋅ 10^−5^) individuals. Moreover, the Halo Effect is significantly stronger for Preoccupied attached individuals, as compared to Fearfully attached individuals (Mann–Whitney U = 1217050.0 Corrected p = 6.841 ⋅ 10^−4^). No significant differences in the strength of the Halo are found between Preoccupied and Securely attached individuals (Mann–Whitney U = 1054499.5, Corrected p = 0.124), Preoccupied and Dismissing individuals (Mann–Whitney U = 740293.5, Corrected p = 0.115) and between Fearfully and Dismissing attached individuals (Mann–Whitney U = 1464726.0, Corrected p = 0.327). A graphical representation of the comparisons is reported in Fig 3, while a representation of the differences in the strength of the Halo Effect before and after the presentation of a prime by condition is reported in Fig 3.

For our third hypothesis H_3_ to be verified, which is that the strength of the Halo after priming is affected by the prime, as well as by the age of presented face, we should find a significant interaction effect between the Age of presented faces (indicated as Age in Table 2). the prime used (indicated as Priming in Table 2), and the time reference that indicates whether data have been collected before or after the presentation of a Prime (indicated as Prime in Table 2). Results of the MLR revealed no significant interaction effect between Age, Priming, and Time (t = 0.397, p = 0.691). A post-hoc analysis revealed that only when primed with a video that promotes Social Distancing, the strength of the Halo Effect significantly increased for children faces (Mann–Whitney U = 54227.5, Corrected p = 3.419 ⋅ 10^−4^) and adults (Mann–Whitney U = 65796.0, Corrected p = 0.013), but not for elderly faces (Elder Mann–Whitney U = 70115.0, Corrected p = 0.501). Significant increases in the strength of the Halo Effect were found for adults participants for both the Neutral (Mann–Whitney U = 55123.5, Corrected p = 0.0145) and Close Contact with others conditions (Mann–Whitney U = 65991.5, Corrected p = 5.148 ⋅ 10^−3^). Differences were not found when participants were shown children or elderly faces after the presentation of a neutral video (Children: Mann–Whitney U = 51817.0, Corrected p = 0.512; Elderly: Mann–Whitney U = 60558.0, Corrected p = 1.016) or a video that promotes Close Contacts with others (Children: Mann–Whitney U = 61543.0, Corrected p = 0.173; Elderly: Mann–Whitney U = 71489.0, Corrected p = 0.918).

For the fourth and last hypothesis H_4_ to be verified, which is that the magnitude of the change after priming is modulated by participants’ attachment styles, we should find a significant interaction effect between Priming, Time, and Attachment Styles. No significant interaction effect between the three has been found (t = 0.497, p = 0.619). Post-hoc analyses revealed that the Halo Effect is significantly stronger after priming a video that promotes Social Distancing for Fearful (Mann–Whitney U = 54834.5 Corrected p = 0.024) and Secure Attached individuals (Mann–Whitney U = 55363.5 Corrected p = 0.021), as well as for Preoccupied attach individuals primed with a video promoting Close Contact with other (Mann–Whitney U = 25356.0 Corrected p = 0.018), but not for the other conditions. Complete details about the post-hoc comparisons are reported in Table 3.

**Table 3:** Average strength of the Halo before and after the presentation of a prime, post-hoc comparison by mean of a Mann–Whitney U test (Bonferroni corrected), and average difference between the strength of the Halo before and after the presentation of a prime (ΔHalo), by condition and attachment style.

Condition	Attach.	Halo			Corr. p	ΔHalo
		Before prime	After Prime	M.W. U		
Social Dist.	Fearful	0.653 ± 0.389	0.704 ± 0.377	54834.5	**0.024** *	0.060 ± 0.372
	Secure	0.701 ± 0.325	0.754 ± 0.295	55363.5	**0.021** *	0.047 ± 0.329
	Preoccupied	0.572 ± 0.450	0.681 ± 0.382	13546.0	0.055	0.111 ± 0.397
	Dismissing	0.669 ± 0.380	0.710 ± 0.366	28036.5	0.719	0.043 ± 0.365
Neutral	Fearful	0.496 ± 0.457	0.569 ± 0.43	74660.0	0.065	0.073 ± 0.481
	Secure	0.707 ± 0.338	0.709 ± 0.35	51596.0	1.175	-0.000 ± 0.375
	Preoccupied	0.735 ± 0.338	0.797 ± 0.295	10539.0	0.131	0.069 ± 0.311
	Dismissing	0.680 ± 0.325	0.700 ± 0.316	10672.0	2.329	0.016 ± 0.310
Close Cont.	Fearful	0.697 ± 0.350	0.684 ± 0.43	63334.0	0.413	-0.011 ± 0.393
	Secure	0.711 ± 0.360	0.729 ± 0.36	30889.0	0.466	0.021 ± 0.326
	Preoccupied	0.597 ± 0.438	0.643 ± 0.476	25356.0	**0.018** *	0.041 ± 0.378
	Dismissing	0.614 ± 0.392	0.661 ± 0.378	33604.0	0.609	0.052 ± 0.337

Further analysis have been conducted to investigate the effect of the Age of presented faces on the strength of the Halo Effect (t = 4.117, p < 0.001). Post-hoc comparisons revealed that the effect is significantly weaker for children’s faces as compared to adults’ (Mann-Whitney U = 2250099.5, Corrected p = 2.153 ⋅ 10^−4^) and elders’ faces (Mann-Whitney U = 2252644.5, Corrected p = 2.744 ⋅ 10^−4^), while no differences were found in the strength of the effects between adults’ and elders’ faces (Mann-Whitney U = 2558181.5, Corrected-p = 1.000).

To investigate in detail the differences between different primes (Condition) and Attachment styles, an analysis of the differences in the strength of the Halo before and after the presentation of a prime, by condition and attachment style, was conducted. It is observed that in all the situations the strength of the Halo increases but in Fearful attached individuals in the Close Contact condition (ΔHalo = -0.011 ± 0.393, Table 3).

## Discussion

The possibility of influencing the strength of the Halo Effect of strangers’ faces puzzled researchers for many years. While attempts at investigating the possibility of manipulating the Halo Effect has been conducted, several studies failed at achieving sufficient power due to a limited sample size [46], or failed in controlling the perceived aesthetic appearance of faces [47]. While the difficulty of controlling the perceived aesthetic appearance of faces seems to suggest a bi-directionality of the Halo Effect, the homogeneity of past works’ participants’ pool may have influenced the collected data [48]. In the current work, we aimed to investigate the possibility of influencing, using an experimental manipulation, the strength of the Halo Effect, considering different characteristics of rated faces, as well as verifying the protective effects of different attachment styles on the magnitude of the changes caused by the experimental manipulation.

In line with a recent work that employed a similar experimental paradigm [21], we found no influence of the interaction between raters’ and rated faces’ gender. As opposed to past works on the Halo Effect, we found that the effect does not seem to be influenced by the possible attraction one has towards the same or opposite gender. This result is a further confirmation of the fact that the Halo Effect is not gender-specific, and when making trustworthiness inferences about others, individuals do not have different strategies for members of the same or different gender. Additionally, an analysis of the effect of the Age of presented faces on the strength of the Halo Effect (t = 4.117, p < 0.001) revealed that the effect is significantly weaker for children’s faces as compared to both adults’ (Mann-Whitney U = 2250099.5, Corrected p = 2.153 ⋅ 10^−4^) and elders’ faces (Mann-Whitney U = 2252644.5, Corrected p = 2.744 ⋅ 10^−4^). No differences were found in the strength of the effects between adults’ and elders’ faces (Mann-Whitney U = 2558181.5, Corrected-p = 1.000). These results are a further confirmation of the specificity of children faces, and of their crucial role in eliciting adults’ responses toward them [21, 49]. Similarly to previous works [21, 50], we found no significant effect of Ethnicity on the strength of the Halo Effect (t = 0.227, Corrected-p = 0.820). One possibility, as indicated by Xu et al. [50], both Asian and White participants employ similar facial cues in their evaluation of attractiveness and trustworthiness of strangers’ faces. Moreover, given the increasing multiculturalism of our society, it is possible that individuals are getting higher exposure to peers of different ethnicities, reducing the impact of the other-race effect [51].

For what concerns the different attachment styles, results highlighted the existence of significant differences in the strength of the Halo Effect accordingly to the attachment styles. More specifically, the relationship between perceived aesthetic appearance and perceived trustworthiness was higher for Securely attached individuals, followed by Dismissing attached individuals and Preoccupied attachment, while the relationship was weaker for Fearfully attached individuals. One possible explanation for this result maybe found in how Secure versus Insecure attached individuals interact with other socially [52]. More specifically, it is possible that Secure individuals rely more on the Halo Effect, as compared to individual with non-secure attachment styles, for which different factors may play a more dominant role in shaping the judgments of strangers’ trustworthiness. For example, previous works found that insecurely attached individuals tend to take a larger interpersonal distance from other, therefore when faces are presented, a same distanca may be more comfortable for securely attached individuals, leading to higher trustworthiness scores [53].

From the visual representation and from the slopes of the linear regression (the stronger the Halo Effect, the closer is the slope of the regression to 1), we can see how Fearful attached individuals are more likely than other individuals to give, at analog levels of aesthetics judgments, lower trustworthiness ratings. This may reflect a protective behavior of Fearfully attached individuals in judging the trustworthiness of strangers, as compared to Securely attached individuals.

These differences may also explain why a limited number of significant differences before and after the presentation of a prime for the different attachment styles (H_4_).

It is possible that the primes we employed in this experiment are not sufficiently influencing participants’ emotions to affect the strong initial differences across differently attached participants. However, looking at the differences before and after the presentation of a prime (Fig 2), significant differences before and after priming for the three conditions were found. While we expected a general decrease in strangers’ trustworthiness after the presentation of a prime promoting social distancing, it is possible that priming Social Distancing increases the participants’ focus on aesthetics aspects to make a stronger assumption about a strangers’ trustworthiness, as compared to priming a neutral or a positive situation, therefore increasing the correlation of the two measures. It is in fact possible that asking participants to focus on stranger’s faces after being primed for social distancing does not elicit an analytic thinking, which has been proved to reduce the strength of the Halo Effect [54], but does instead force viewers to focus more on the aesthetic appearance of a stranger to identify possible cues that could undermine their trustworthiness, such as tattoos [55], or symptoms related to the coronavirus.

To put the results in the context of this works’ aim and hypothesis, not all our hypotheses have been confirmed. For what concerns gender differences, we found no proof in support of the existence of a significant interaction between raters’ and ratees’ gender. Similar to our previous work [21], results do not support the idea that the Halo Effect is stronger for opposite gender faces. In light of this, we can further confirm the universality of the effect across Gender, as well as across Ethnic groups (t = 0.227, p = 0.82, Table 2). Moreover, as with results of past works, we found significant differences in the strength of the Halo Effect between children’s and adults’ faces, further supporting the specificity of children’s faces. For what concerns the possibility of manipulating the strength of the Halo Effect in a controlled situation, we found that priming participants with information about Social Distancing or Close Contact with others induced significant changes in the strength of the Halo Effect, but as opposed to our predictions, changes are in the same directions (stronger Halo Effect after priming). Therefore we can confirm the strength of the Halo Effect Aesthetics × Trustworthiness can be experimentally manipulated. Finally, for what concerns the differences in the strength of the Halo Effect in participants with different attachment styles, results suggest that while the attachment style seems to have an influence on the strength of the Halo Effect influence on a Prime, limited significant differences were found before and after a prime is presented. This suggests that none of the styles is causing a higher magnitude of changes in the aesthetics and trustworthiness ratings given to strangers’ faces.

Before concluding, it is worth acknowledging the limitations of the current work. First, despite our best attempts at obtaining a balanced sample in terms of ethnicity and gender, the number of participants per group is not identical. This is especially so for the Asian sample, where the number of Women participants outnumbers the number of Men participants. To avoid overpowering the analysis, and to keep faith with our pre-registered methods, we have decided to avoid adding Men participants to balance the number of participants in each cell. Because of this design choice, results of this work may cause our results to be more generalizable to Women than Men individuals, especially for what concerns Asian individuals. Moreover, there seems to be a noteworthy age difference between Asian and White participants. While this may be partially due to the procedure we employed to recruit participants, given the age range participants were asked to rate, we believe the influence of participants’ age to be minimal. Future works should attempt at obtaining a more balanced sample in terms of age and gender by enrolling a specified number of participants per group, and not by inviting volunteers to participate freely in multiple media, and by taking into account previous or current neuropsychiatric conditions. Second, the number of stimuli used in this work—less than a hundred different samples—is quite limited. Future works should try to obtain ratings of a bigger number of faces, to allow the analysis of the traits using different analytic methods (e.g. Neural Networks). Finally, only three videos have been used as prime. Future works should try to employ a higher number of primes, to better investigate the effects of different prime on the Strength of the Halo Effect.

## Conclusions

In this work, we verified the influence of gender on the strength of the Halo Effect, the possibility of affecting the strength of the Halo using experimental manipulation, and differences in strength of the Halo across individuals with different attachment styles. While we found no significant differences in the strength of the Halo when participants were rating strangers of the same versus different gender, we have found significant differences in the strength of the effect in the ratings of children faces after participants have been primed with a video promoting Social Distancing. Moreover, while we found significant differences in the strength of the Halo for participants who presented different attachment styles, the latter does not seems to influence the level of change participants’ presented after the priming procedure has been conducted. Our results provide preliminary insights on the possibility of affecting the strength of the relationship between perceived Aesthetics and perceived trustworthiness using manipulation and help shed the light on how the attachment style influences individuals’ impression formation. Future works should verify how different primes affects the strength of the Halo, and the possibility of exploiting situations to increase (or decrease) someone’s perceived trustworthiness.

## Supporting information

S1 Table Multiple Linear Regression results. Complete summary of main and interaction effects analysis conducted via Multiple Linear Regression.
(PDF)
