# Supplementary material for: Influences of Social Distancing and attachment styles on the strength of the Halo Effect
Source: PLoS One. 2021 Aug 26;16(8):e0256364. doi: 10.1371/journal.pone.0256364 (PMC8390124; doi:10.1371/journal.pone.0256364)
Supplement: S1 Table — Complete summary of main and interaction effects analysis conducted via Multiple Linear Regression. (PDF) [file pone.0256364.s001.pdf]

S1 Table. Multiple Linear Regression results. Complete summary of main and interaction effects analysis conducted via Multiple Linear Regression.

	Coeff	Std. Error	t	p-value	95% CI
Intercept	0.6562	0.007	93.044	¡ 0.001*	[0.642; 0.67]
Age	0.0357	0.009	4.117	¡ 0.001*	[0.019; 0.053]
Attachment Style	0.0096	0.004	2.224	0.026*	[0.001; 0.018]
Ethnicity	0.0016	0.007	0.227	0.82	[-0.012; 0.015]
Gender	-0.0012	0.007	-0.169	0.866	[-0.015; 0.013]
Priming	0.001	0.009	0.116	0.908	[-0.016; 0.018]
Time	0.0141	0.007	2.006	0.045*	[0; 0.028]
Age × Attachment Style	0.0057	0.005	1.074	0.283	[-0.005; 0.016]
Age × Ethnicity	-0.0021	0.009	-0.239	0.811	[-0.019; 0.015]
Age × Gender	0.006	0.009	0.691	0.49	[-0.011; 0.023]
Age × Priming	0.0143	0.011	1.323	0.186	[-0.007; 0.035]
Age × Time	-0.0066	0.009	-0.756	0.45	[-0.024; 0.01]
Ethnicity × Attachment Style	-0.002	0.004	-0.469	0.639	[-0.011; 0.006]
Ethnicity × Gender	0.0049	0.007	0.698	0.485	[-0.009; 0.019]
Gender × Attachment Style	-0.0025	0.004	-0.568	0.57	[-0.011; 0.006]
Priming × Attachment Style	-0.0084	0.005	-1.606	0.108	[-0.019; 0.002]
Priming × Ethnicity	0.0063	0.009	0.717	0.474	[-0.011; 0.023]
Priming × Gender	-0.0091	0.009	-1.034	0.301	[-0.026; 0.008]
Priming × Time	-0.0132	0.009	-1.51	0.131	[-0.03; 0.004]
Time × Attachment Style	0.0047	0.004	1.078	0.281	[-0.004; 0.013]
Time × Ethnicity	0.001	0.007	0.147	0.883	[-0.013; 0.015]
Time × Gender	0.0015	0.007	0.211	0.833	[-0.012; 0.015]
Age × Ethnicity × Attachment Style	0.0057	0.005	1.069	0.285	[-0.005; 0.016]
Age × Ethnicity × Gender	0.0019	0.009	0.223	0.823	[-0.015; 0.019]
Age × Gender × Attachment Style	0.0014	0.005	0.259	0.796	[-0.009; 0.012]
Age × Priming × Attachment Style	-0.0084	0.006	-1.31	0.19	[-0.021; 0.004]
Age × Priming × Ethnicity	0.0082	0.011	0.756	0.45	[-0.013; 0.029]
Age × Priming × Gender	0.0008	0.011	0.076	0.939	[-0.02; 0.022]
Age × Priming × Time	0.0043	0.011	0.397	0.691	[-0.017; 0.025]
Age × Time × Attachment Style	-0.0032	0.005	-0.592	0.554	[-0.014; 0.007]
Age × Time × Ethnicity	-0.0022	0.009	-0.25	0.803	[-0.019; 0.015]
Age × Time × Gender	-0.0031	0.009	-0.362	0.718	[-0.02; 0.014]
Ethnicity × Gender × Attachment Style	-0.0011	0.004	-0.26	0.795	[-0.01; 0.007]
Priming × Ethnicity × Attachment Style	-0.0048	0.005	-0.924	0.355	[-0.015; 0.005]
Priming × Ethnicity × Gender	-0.0004	0.009	-0.044	0.965	[-0.018; 0.017]
Priming × Gender × Attachment Style	0.0092	0.005	1.775	0.076	[-0.001; 0.019]
Priming × Time × Attachment Style	0.0026	0.005	0.497	0.619	[-0.008; 0.013]
Priming × Time × Ethnicity	0.0005	0.009	0.059	0.953	[-0.017; 0.018]
Priming × Time × Gender	-0.0013	0.009	-0.152	0.879	[-0.019; 0.016]
Time × Ethnicity × Attachment Style	-0.0003	0.004	-0.063	0.95	[-0.009; 0.008]
Time × Ethnicity × Gender	0.007	0.007	0.998	0.318	[-0.007; 0.021]
Time × Gender × Attachment Style	-0.0042	0.004	-0.958	0.338	[-0.013; 0.004]
Age × Ethnicity × Gender × Attachment Style	0.0001	0.005	0.023	0.982	[-0.01; 0.011]
Age × Priming × Ethnicity × Attachment Style	-0.0025	0.006	-0.391	0.696	[-0.015; 0.01]
Age × Priming × Ethnicity × Gender	0.0084	0.011	0.776	0.438	[-0.013; 0.03]
Age × Priming × Gender × Attachment Style	0.0008	0.006	0.124	0.901	[-0.012; 0.013]
Age × Priming × Time × Attachment Style	0.0021	0.006	0.336	0.737	[-0.01; 0.015]
Age × Priming × Time × Ethnicity	-0.0014	0.011	-0.126	0.9	[-0.023; 0.02]
Age × Priming × Time × Gender	0.0003	0.011	0.028	0.978	[-0.021; 0.021]
Age × Time × Ethnicity × Attachment Style	0.0019	0.005	0.357	0.721	[-0.009; 0.012]
Age × Time × Ethnicity × Gender	-0.004	0.009	-0.457	0.648	[-0.021; 0.013]
Age × Time × Gender × Attachment Style	0.0029	0.005	0.535	0.592	[-0.008; 0.013]
Priming × Ethnicity × Gender × Attachment Style	-0.0017	0.005	-0.329	0.742	[-0.012; 0.008]
Priming × Time × Ethnicity × Attachment Style	0.0027	0.005	0.51	0.61	[-0.008; 0.013]
Priming × Time × Ethnicity × Gender	0.0046	0.009	0.519	0.604	[-0.013; 0.022]
Priming × Time × Gender × Attachment Style	-0.0031	0.005	-0.598	0.55	[-0.013; 0.007]

	Coeff	Std. Error	t	p-value	95% CI
Time × Ethnicity × Gender × Attachment Style	-0.0042	0.004	-0.966	0.334	[-0.013; 0.004]
Age × Priming × Ethnicity × Gender × Attachment Style	-0.0048	0.006	-0.747	0.455	[-0.017; 0.008]
Age × Priming × Time × Ethnicity × Attachment Style	0.0018	0.006	0.279	0.781	[-0.011; 0.014]
Age × Priming × Time × Ethnicity × Gender	-0.0006	0.011	-0.055	0.956	[-0.022; 0.021]
Age × Priming × Time × Gender × Attachment Style	-0.0039	0.006	-0.605	0.545	[-0.016; 0.009]
Age × Time × Ethnicity × Gender × Attachment Style	0.0002	0.005	0.028	0.977	[-0.01; 0.011]
Priming × Time × Ethnicity × Gender × Attachment Style	-0.0047	0.005	-0.91	0.363	[-0.015; 0.005]
Age × Priming × Time × Ethnicity × Gender × Attachment Style	0.0003	0.006	0.053	0.957	[-0.012; 0.013]

* $p < 0.05$
